# Evaluation of health effect on workers exposed to methyl bromide with prefrontal event-related potential

**DOI:** 10.1371/journal.pone.0328580

**Published:** 2025-07-30

**Authors:** Jungmi Choi, Wonseok Cha, Young-Seoub Hong, Min-Goo Park

**Affiliations:** 1 Human Anti-Aging Standards Research Institute, Uiryeong-gun, Gyeongsangnam-do, Republic of Korea; 2 Department of Preventive Medicine, College of Medicine, Dong-A University, Busan, Republic of Korea; 3 Department of Bioenvironmental Chemistry, Jeonbuk National University, Jeonju, Republic of Korea; Central Food Technological Research Institute CSIR, INDIA

## Abstract

Methyl bromide (MB) is a potent fumigant used to control pests in soil and agricultural products. As an ozone-depleting substance, MB has been largely replaced by safer alternatives. MB is highly toxic to humans and has been shown to adversely affect asymptomatic workers’ central and autonomic nervous systems and vascular health. However, its impact on perceptual and cognitive abilities remains underexplored. In this study, we examined the effects of MB exposure on cognitive functions in asymptomatic workers. Event-related potential (ERP) indices, which reflect perceptual and cognitive processes, and urinary bromide ion (Br^-^) concentrations were assessed in 32 fumigators (study group) and 18 inspectors (control group) before and after fumigation. Post-work ERP latency and amplitude changes in inspectors were significant (*P* < 0.01), similar to those observed in healthy individuals. In contrast, ERP changes in fumigators were not significant compared to pre-work values; this suggests that MB negatively impacts cognitive health. Additionally, Br^‑^ levels in fumigators rose sharply after work (*P* < 0.001), while inspectors showed no such increase. The elevated Br^-^ levels and nonenhanced ERP indices in fumigators after MB exposure indicate adverse health effects despite the absence of symptoms.

## 1. Introduction

Methyl bromide (MB), formerly widely used as a fumigant in agriculture to control pests, poses significant environmental hazards [1–3]. As an ozone-depleting substance, it contributes to the depletion of the ozone layer, increasing the risk of adverse ultraviolet radiation arriving the surface of the Earth [4]. In response, the Montreal Protocol was established by the United Nations Environmental Program to phase out MB, with exemptions for critical uses such as quarantine purposes [4]. Countries under the Protocol have explored alternative pest control methods to reduce environmental damage [5]. Adopting safer alternatives is crucial for protecting the ozone layer and mitigating climate change [6].

Besides being a substance that depletes ozone, MB is a highly toxic pesticide that causes significant risks to fumigators and related workers [7–11]. The main route of MB exposure is through inhalation [12]. When MB is absorbed into the body, it has the potential to induce neurological disorders, lung injury, vision impairments, cytotoxicity, genotoxicity, and various other harmful effects [7,13,14]. Neurotoxicity is recognized as the most common exposure outcome in humans and animals [15]. Notably, symptoms such as nausea, vomiting, dizziness, tremors, and convulsions have been reported in cases of MB intoxication. These symptoms were linked to acetylcholinesterase, a neurotransmitter involved in the autonomic nervous system [16]. Moreover, several studies have reported that the asymptomatic workers exposed to MB had negative impacts on the central and autonomic nervous systems [17,18] and vascular health [19].

Biological monitoring traditionally involves analyzing MB or its metabolites in blood, urine, or breath. Elevated bromide ion (Br^-^) levels in fumigator urine can indicate exposure, though symptoms of poisoning may not always be apparent [20,21]. This variation in symptom manifestation among individuals could explain the discrepancy [21]. Therefore, we used electroencephalography (EEG) and heart rate variability as sensitive indicators of nervous system function, confirming damage to both central and autonomic systems in exposed workers [17,18]. Second-derivative photoplethysmography was a biomarker for assessing vascular health, revealing negative effects on vascular health [19]. However, the impact of MB fumigation on cognitive functions related to perception remains unexplored, and effective biomarkers for evaluating perceptual function in MB-exposed workers have not yet been identified.

EEG captures electrical signals neurons produce in the brain’s outer layer [22]. When many neurons fire simultaneously, they generate postsynaptic potentials detectable from the scalp. EEG data analysis can reveal normal brain function and abnormal neural activity, such as those seen in MB-exposed workers. This non-invasive technique is relatively safe, fast, cost-effective, widely accessible, and allows repeated measurements in high-risk populations [23]. Event-related potentials (ERPs) are small voltage changes in the EEG signal in response to specific stimuli [24,25]. These voltage peaks are time-locked to events and reflect the brain’s sensory, perceptual, and cognitive functions. ERPs thus provide insights into both physiological and pathological aspects of cognitive function.

Numerous studies have validated ERP as a reliable method for assessing cognitive impairment, such as Alzheimer’s disease. ERP latencies are potential indicators for screening individuals with mild cognitive impairment (MCI) or Alzheimer’s disease [26–28]. A delayed ERP response often signifies reduced cognitive ability to process and recognize stimuli, with individuals with MCI or Alzheimer’s disease showing prolonged peak latencies compared to cognitively normal individuals [26–28]. Previous studies have demonstrated the use of ERP to evaluate cognitive decline in patients with Alzheimer’s [29] and its correlation with Mini-Mental Status Examination (MMSE) scores [30], a common dementia screening tool. Our study utilized these tools and protocols to evaluate cognitive deterioration in MB-exposed workers.

In this study, we aimed to assess the cognitive effects of occupational MB exposure in workers. ERP signals were measured before and after fumigation, and conventional biomarker analysis of Br^-^ in urine was carried out to quantify MB exposure.

## 2. Materials and methods

### Participants

This study included 44 participants employed by pest control companies using MB, all of whom were registered with the Animal and Plant Quarantine Agency (APQA) in Korea [31]. The control group comprised 20 public officials from the Youngnam Regional Office of the APQA in Busan, Korea, who supervised the MB operations.

From February 1st to August 31st, 2019, ERP signals were evaluated for one or two individuals from each group on the day of MB fumigation. All participants underwent ERP screening by licensed clinical nurses and urinary Br^-^ levels were measured before and after fumigation. The fumigators purchased MB (Young IL MB) from Nong Hyup Chemical Co., Ltd. The fumigation was carried out at Busan port to control pests in oranges or wood loaded in containers or tarpaulins. Fumigators were responsible for sealing containers, measuring MB concentrations, applying MB, and ventilating the area post-application.

In contrast, quarantine inspectors supervised the fumigation process but had minimal direct MB exposure due to their distinct job roles. The procedure was followed according to the guidelines of APQA [32]. For additional details on participant roles and ERP measurement procedures, refer to Supplementary Table S1 in S1 File.

This observational study, part of the APQA Plant Quarantine Technology Development Program, was approved by the Institutional Review Board of Dong-A University (IRB number: 2-1040709-AB-N-01-201806-BR-004-04). The board approved the research plan, including the analysis and use of urinary Br^-^ ions and EEG data from all participants. All participants provided written informed consent to participate and analyze collected data.

### Demographic information

Table 1 summarizes the demographic details and initial characteristics of the study participants. The fumigation group was predominantly male and older than the inspection group. On test days, gas masks were used more frequently in the fumigation group than in the inspection group. However, other factors, including work duration, smoking, and alcohol consumption, did not show a significant difference between the two groups.

**Table 1:** Demographic information.

Demographic variable	Fumigator	Inspector	*P*-value
Sex: Male	32 (100%)	12 (66.6%)	<0.001
Age (years)	42.97 ± 9.91	36.28 ± 11.08	0.033
Alcohol: Yes	31 (96.9%)	16 (88.9%)	0.254
Smoking: Yes	13 (41.3%)	4 (22.2%)	0.187
Duration of work (years)	10.06 ± 9.24	6.17 ± 7.92	0.139
Gas mask^1^ use on test day: Yes	32 (100%)	11 (61.1%)	<0.001

The data are summarized as the mean ± SD for continuous variables (age and duration of work) and the frequency and proportion for categorical variables (sex, alcohol, smoking, and gas mask use on the test day). *P*-values were derived from the independent t-test for continuous variables and Fisher’s exact test for categorical variables.

^1^Gas masks used by participants during MB fumigation work were attached with a canister for air purification.

MB, methyl bromide; SD, standard deviation.

### Analysis of Br^-^ in urine

Urine samples were collected according to guidelines to avoid contamination [33]. After discarding the initial portion, more than 10 mL of midstream urine was collected using a specified urine cup (Qorpak PLC-03701 Natural Polypropylene Jar with 58–400 White Polypropylene Unlined Cap, 120 mL) and stored at 4 °C until transport for testing. Each 5 mL sample was transferred into specialized conical tubes (CELLTREAT 229412 Centrifuge Tube, 15 mL, polypropylene) and stored in a freezer at −80 °C for Br^-^ concentration analysis. The mobile phase consisted of 18 mM nitric acid (electronic grade) and 34 mM ammonium hydroxide (Sigma-Aldrich, USA). A standard bromide solution (1,000 mg/L; Sigma-Aldrich, USA) was used to construct the calibration curve. Urine samples were diluted 20-fold with deionized water and filtered using a 0.45 μm filter before analysis. Br^-^ levels in urine were determined using a high-performance liquid chromatography-inductively coupled plasma mass spectrometer (HPLC/ICP-MS) system, including an Agilent Technologies 1260 Series HPLC and an Agilent Technologies 7700 Series ICP-MS in a laboratory at Dong-A University. Br^-^ in μg/mg creatinine was calculated by dividing Br^-^ concentration by creatinine concentration and multiplying 1000. The details are described in Table 2.

**Table 2:** HPLC/ICP-MS parameters and settings.

Parameter	Setting
**HPLC parameters and settings**	
Instrument	Agilent Technologies 1260
Analytical column	Hamilton PRP-X100, 5 μm
Column dimensions	4.6 mm × 250 mm
Pump flow	1.5 mL/min
Injection volume	30 μL
Mobile phase	34 mM Ammonium hydroxide
	18 mM Nitric acid
	pH = 9.3
**ICP-MS parameters and settings**	
Instrument	Agilent Technologies 7700 series
RF power	1,550 W
RF matching	1.80 V
Carrier gas flow rate	1.05 L/min
Sampler and skimmer cones	Nickel
Spray chamber temp	2°C
He flow rate	4.3 mL/min
Nebulizer	Micromist nebulizer

### ERP measurements

ERPs were recorded as EEG responses to repetitive auditory pure tones over 8 min at the Youngnam Regional Office in Busan. The stimuli consisted of eight pure tones (125, 250, 500, 750, 1,500, 2,000, 3,000, and 4,000 Hz), each presented with equal probability [29,30]. The tones were delivered in a pseudo-random sequence to prevent consecutive tones of the same frequency. Participants were instructed to passively listen to the tones; 480 stimuli were delivered binaurally through earphones at a volume of 70 dB SPL. Each tone lasted 50 ms, with rise and fall times of 1 ms, and the interval between stimuli was 1 s. During the test, participants were seated comfortably in a well-lit office room.

For collecting the EEG responses, non-invasive monopolar scalp electrodes recorded brain activity from two prefrontal regions (Fp1 and Fp2 in the International 10/20 system), with the right earlobe as a reference. NeuroNicle amplifiers (LAXTHA Inc., Korea) featured a bandpass filter from 3 to 43 Hz and an input range of ±393 µV, with input noise below 0.6 µVrms. Digital filters used included: (1) a 2nd-order band-stop filter at 55 and 65 Hz, (2) a 1st-order high-pass filter at 2.6 Hz, and (3) an 8th-order low-pass filter at 43 Hz. Electrode contact impedances were kept below 10 kΩ, and all data were digitized in continuous recording mode.

To minimize ocular, muscular, and other artifacts, an operator monitored both the subject and EEG traces, guiding the subject to keep their eyes closed and muscles relaxed. Artifacts were not discarded, but data were assessed for contamination from muscle and eye movements in the (Fp1, Fp2) prefrontal EEG.

**Fig 1:**
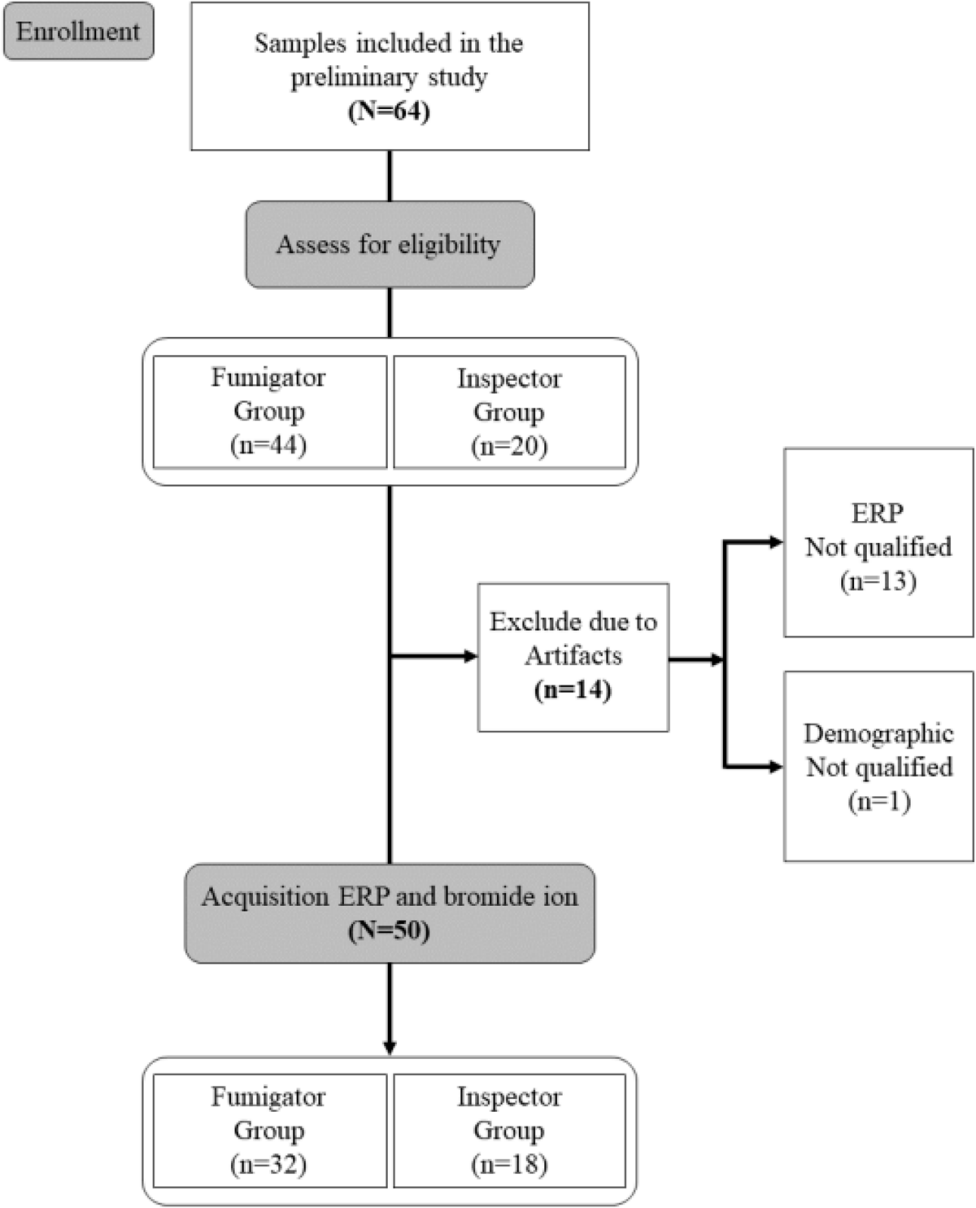
Consolidated Standards of Reporting Trials (CONSORT) diagram illustrating enrollment and exclusion criteria for this study.

EEG data were initially checked for significant artifacts, with 14 of 64 recordings showing more than 10% of epochs exceeding 200 µV, a standard threshold for detecting significant artifacts [34], leading to their rejection. Fig 1 illustrates this study’s consolidated reporting trial standards (CONSORT) diagram.

### ERP variables and computation

ERP markers were obtained using the conventional ensemble averaging method with stimuli. ERP variables, reflecting exogenous sensory components, primarily depend on stimulus physical parameters but can also be influenced by cognitive processes [30]. Five variables were examined: average voltage peak (amplitude), average response time, amplitude deviation, response time deviation, and the amplitude difference from center to edge. The voltage peak, representing the highest amplitude of the ERP signal, was relatively insensitive for detecting dementia changes [30,35]. The response time, or latency, to show the first positive peak relative to stimulus onset can be delayed in patients [30,36].

ERPs are relevant for investigating synaptic impairments since brain disorders like Alzheimer’s disease (AD) are fundamentally linked to synaptic plasticity dysfunction, and ERP peaks are direct reflections of synaptic activity [37]. Early ERP components peak within 250 ms of stimulus onset and primarily capture sensory processes and stimulus attributes. Studies have observed abnormalities in AD, such as delayed latencies and reduced amplitudes of these early ERP components. Specifically, delayed latency of the P200 component has been reported, suggesting that early sensory-cognitive processes may be compromised in AD [38].

Additionally, our previous study, which employed the same ERP protocol as the current one, demonstrated that the predictive value of the P200 component for pre-dementia stages of AD improves when combined with biomarkers such as amyloid-beta levels and metrics like slowed EEG oscillations during eye-closed resting states [29,39]. ERP peaks occurring around 200 ms post-stimulus are associated with automatic cognitive processes, including stimulus discrimination and evaluation, with these changes often emerging early in the progression of the disease. Furthermore, early ERP amplitude is more sensitive to the early stages of AD than P300 latency [40].

### Statistical analysis

ERP voltage peaks, response times, and urinary Br^-^ concentrations are reported as means and standard errors (SE). Independent t-tests compared baseline characteristics for continuous variables, while chi-square tests assessed categorical variables. Independent t-tests compared ERP and Br^-^ levels between the study and control groups before and after fumigation. Paired *t*-tests evaluated ERP and Br level changes within each group before and after fumigation. Pearson’s correlation and regression analyses examined the relationship between urinary Br^-^ concentrations and ERP indices across all participants. Data analysis was performed using SPSS version 23, with significance at P < 0.05.

## 3. Results

### Comparison of ERP indices and urinary Br^-^ concentration before and after MB work

**Fig 2:**
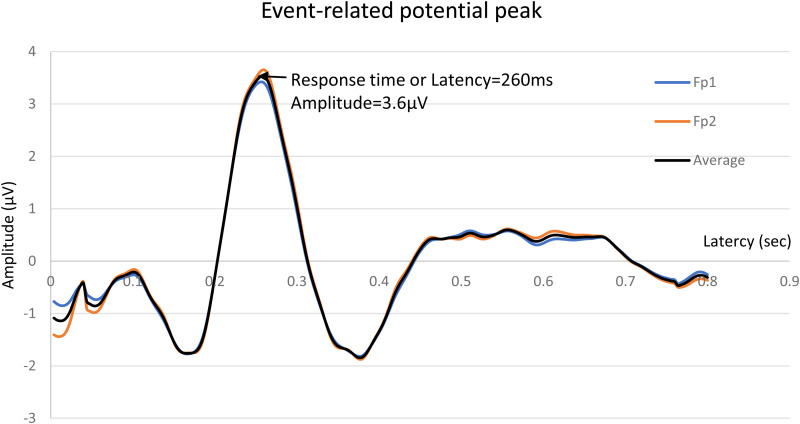
Event-related potential (ERP) peaks measured in one participant. Fp1: left prefrontal lobe, Fp2: right prefrontal lobe, Average: mean value of Fp1 and Fp2.

Fig 2 shows ERP responses to auditory stimuli in workers.

**Fig 3:**
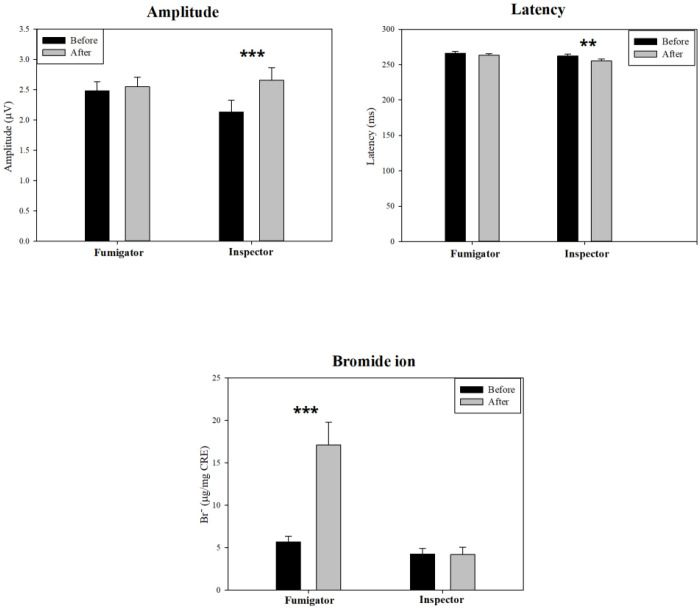
Changes in Event-related potential (ERP) indices and Br^‑^ levels of fumigators and inspectors before and after fumigation work. ^*^P < 0.05, ^**^P < 0.01, and ^***^P < 0.001. Br^‑^, bromide ion. The amplitude and latency of ERPs in inspectors varied after work, whereas the ERPs of fumigators did not show significant variation. Br⁻ levels increased in fumigators but remained unchanged in inspectors.

Table 3 and Fig 3 summarize changes in ERP indices and urinary Br^-^ concentrations over time for each group. Fig 3 displays the differences in the indices between before and after fumigation for each group, based on Table 2. Results indicated that after work, inspectors experienced significant changes in ERP latency and amplitude compared to their pre-work values (*P* < 0.01). Specifically, latency decreased from 262 to 255 ms, while amplitude increased from 2.13 to 2.66 µV. This change could be attributed to circadian fluctuations, as ERP tends to be enhanced in the afternoon compared to the morning [41]. In contrast, fumigators did not show significant changes in ERP levels before and after work, possibly due to the attenuation of enhanced ERP values in the afternoon.

**Table 3:** Urinary bromide ion concentrations (μg/mg CRE) and ERP indices of fumigators and inspectors before and after fumigation work.

Index	Group	Before	After	*P* ^1^
Br-	Fumigator (n = 32)	5.69 ± 0.66	17.11 ± 2.69	<0.001
(μg/mg CRE)	Inspector (n = 18)	4.27 ± 0.70	4.19 ± 0.87	0.918
	*P* ^2^	0.166	<0.001	–
ERP latency	Fumigator (n = 32)	266.00 ± 2.58	263.13 ± 2.53	0.350
(ms)	Inspector (n = 18)	262.44 ± 2.74	255.22 ± 2.88	<0.001
	*P* ^2^	0.380	0.055	–
ERP amplitude	Fumigator (n = 32)	2.48 ± 0.15	2.55 ± 0.15	0.505
(µV)	Inspector (n = 18)	2.13 ± 0.19	2.66 ± 0.21	0.001
	*P* ^2^	0.155	0.681	–

Urinary bromide ion concentrations and ERP indices are expressed as mean ± SE.

^1^*P*-values were indicated based on paired t-test.

^2^*P*-values were indicated based on independent t-test.

Mean urinary Br^-^ concentrations before work were 5.69 μg/mg creatinine (CRE) for fumigators and 4.27 μg/mg CRE for inspectors, with no significant difference between the groups (*P* = 0.166). After work, urinary Br^-^ concentrations increased to 17.11 μg/mg CRE for fumigators and 4.19 μg/mg CRE for inspectors, showing a significant difference between the groups (*P* < 0.001). The average urinary Br- concentration in fumigators increased significantly after work compared to before work (*P* < 0.001), whereas no significant change was observed in inspectors. The LOD and LOQ for this method were 0.935 ug/L and 2.979 ug/L for Br^-^, respectively. The recovery rate for spiked samples of 20 ug/L was 96.84% based on seven replications. A representative chromatogram is shown in Fig S1 in S1 File. The raw data of ERP indices and urinary Br^-^ is provided in Tables S2 and S3 in S1 File.

### Correlation between Br- levels and ERP indices in all individuals

**Fig 4:**
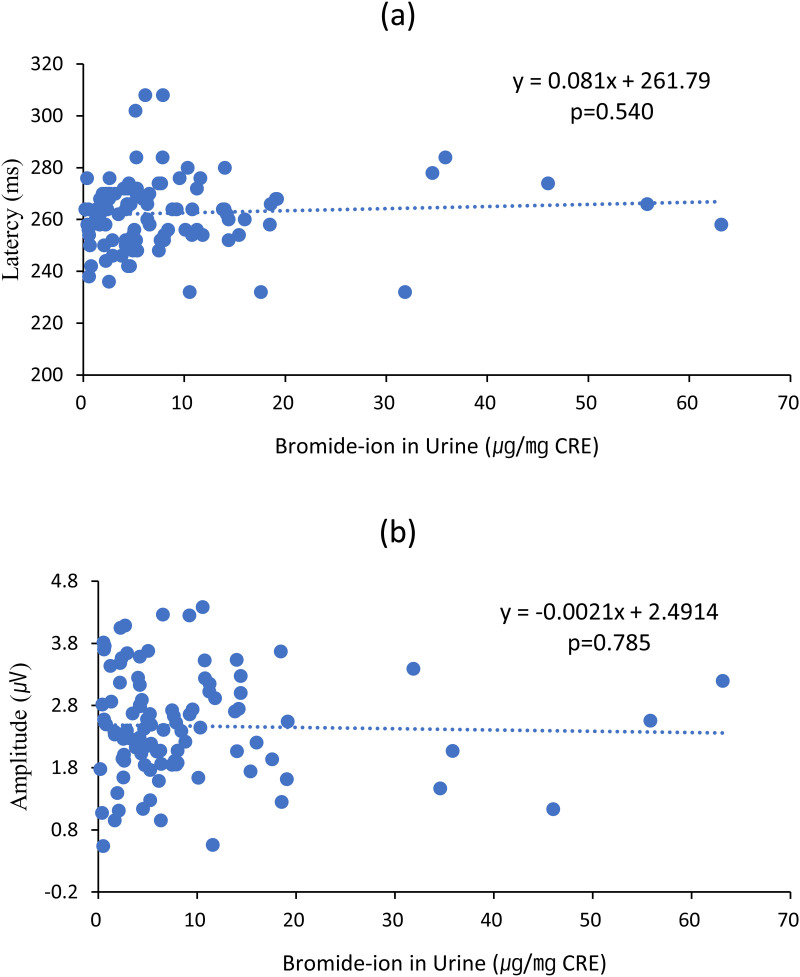
Correlation between urinary Br- levels and ERP indices in all participants before (n = 50) and after (n = 50) work. **(a)** Latency increased with higher Br⁻ levels, while (b) amplitude decreased as Br⁻ levels increased.

The correlations between urinary Br^-^ levels and ERP indices were analyzed for all 50 participants before and after work. The analysis revealed a positive correlation (Pearson’s coefficient = 0.062) between Br^-^ levels and latency values and a negative correlation (Pearson’s coefficient = −0.028) between Br^-^ levels and amplitude values, although these correlations were not significant. Linear regression analysis showed an increase in latency (y = 0.081x − 261.79) and a decrease in amplitude (y = −0.0021x + 2.4914) with Br^-^ levels, but these results were not significant. Complete correlation and regression analyses are presented in Table 4 and Fig 4. Fig 4 shows the trend equations in latency and amplitude, based on Table 4.

**Table 4:** Correlations of urinary Br^-^ levels, and ERP indices among all participants before and after work.

–	Latency	Amplitude
Correlation	0.062	−0.028
*P*-value	0.540	0.785
d.f.	98	98

The number of participants was 50 before and after work. Pearson’s correlation analysis examined the relationship between urinary Br^-^ concentrations and ERP indices across all participants.

## 4. Discussion

ERP analysis has been widely employed to quantitatively assess cognitive impairment related to conditions such as dementia and Alzheimer’s disease [24,29,30,36,38,39]. Specifically, prolonged ERP latencies are observed in neurodegenerative states [29], and ERPs have been correlated with MMSE scores across different age groups [30]. In the present study, ERP indices were used to evaluate the effects of MB on cognitive function. The mean ERP indices in the quarantine group post-MB exposure significantly differed from pre-exposure values: response time to stimuli accelerated, and amplitude increased notably (Table 3 and Fig 3). In contrast, the ERP indices of fumigators in the control group remained unchanged (Table 3 and Fig 2).

Few studies have examined circadian fluctuations in biosignals. One study found the highest perceptual ability for tactile sensing at 18:00 compared to 12:00 and 09:00, as measured using a biological monitoring sensor [42]. Another study found that adolescent ERP responses in the afternoon exhibit faster response times and higher amplitudes compared to those in the morning [41]. The amplitude and latency in the inspector group (control group) mirrored the patterns observed in earlier studies. Conversely, the lack of variance in fumigator ERPs might be due to the degradation of response time and amplitude in the afternoon, potentially influenced by sensory perception changes caused by MB exposure.

An HPLC/ICP-MS method, adapted from traditional ICP-MS techniques [43,44], was used in this study. Earlier research has examined Br^-^ levels in biological samples gathered from MB workers. One study reported mean urinary Br^-^ concentrations of 9.1 mg/L in 251 MB workers compared to 6.3 mg/L in 379 non-MB workers [20]. Another study found the synthesis group at an MB manufacturing plant had a median Br- level of 13.0 μg/mg CRE over 17 years. In contrast, another group had a median level of 7.2 μg/mg CRE [21]. This study reported an average post-work urinary Br^-^ concentration of 17.11 μg/mg CRE in 32 fumigation workers, a significant increase from the pre-work level of 5.69 μg/mg CRE, which was approximately three times higher than the pre-work level. There was no significant change in Br^-^ levels in inspectors.

Pesticides controlling insects or fungi have been reported to possess neurotoxic properties [45,46]. MB is one pesticide that has shown negative effects on fumigators’ central nervous systems, as assessed by electroencephalogram (EEG) analysis [17]. This conclusion is supported by a significant relationship between EEG patterns and bromide (Br⁻) levels. ERPs are indirectly related to EEG because ERPs are derived from EEG measurements [39]. Alzheimer’s patients exhibit longer ERP latencies and delayed median EEG frequencies [29].

In this study, the relationship between urinary Br⁻ concentrations and ERP latencies or amplitudes was not statistically significant. However, a positive correlation was observed between urinary Br⁻ levels and the latency of all ERP components (Table 4 and Fig 4). Additionally, ERP amplitudes showed a negative correlation with urinary Br⁻ levels (Table 4 and Fig 4), a trend resembling that observed in Alzheimer’s patients. Notably, a previous study with a larger sample size of 44 fumigators and 20 inspectors revealed a significant correlation between Br⁻ levels and EEG changes [17], which are indirectly linked to ERP patterns [29].

Furthermore, considering the workplace environment in this study involved exposure to MB concentrations near 1,000 ppm—substantially exceeding the permissible exposure limit of 1 ppm [47] —it can be inferred that elevated Br⁻ levels could influence ERP indices. These findings suggest that ERP measurements may be useful for assessing cognitive functional decline and predicting disease risk associated with MB exposure.

This study was conducted on fumigators and inspectors, who would not be typically considered standard study groups due to their work in a port area with restricted access. A key accomplishment of this study is its contribution to understanding the health impacts for fumigators who exhibit no symptoms of toxicity despite being exposed to MB in their work. However, a limitation of the study was the insufficient sample size for the study and control group, as participants were selected from 76 fumigators and 36 inspectors registered with the APQA in the Busan Port area, compounded by the restricted access to this population. Although previous research with a similar number of participants was accepted [17], the present study still included a relatively small sample size of 32 fumigators and 18 inspectors, which led to insignificant correlations between urinary Br^-^ concentrations and ERP indices. Moreover, MB exposure was primarily associated with acute health effects. Future research should involve a larger sample size to evaluate the long-term health effects of MB exposure.

Despite these limitations, the study provided evidence of adverse health effects in workers exposed to MB, including increased urinary Br^-^ levels and changes in ERP indices post-exposure. These findings have practical implications for enhancing fumigation procedures, mitigating hazards, and safeguarding employee health. They also support efforts to limit MB use and promote alternative methods.

## 5. Conclusions

We measured ERP indices and urinary Br^-^ concentrations in 32 fumigators (study group) and 18 inspectors (control group) before and after fumigation. These indices assess perceptual and cognitive abilities. While the post-work latency and amplitude of ERP in inspectors showed significant changes, similar to those observed in healthy individuals, no significant changes were noted in the ERP indices of fumigators compared to their pre-work values; this suggests that MB negatively affects cognitive health. Additionally, Br^-^ levels in fumigators increased sharply after work, whereas no significant difference was observed in inspectors. These findings indicate that both Br^-^ levels and ERP indices were altered after MB exposure in the fumigator group, resulting in negative health effects, even in the absence of overt symptoms. These findings will promote stricter regulations for worker safety, requiring workers to maintain a safe distance from fumigation chambers and wait sufficient time to release MB. Furthermore, the use of MB for fumigating commodities will be gradually phased out and reduced while efforts to develop alternatives to MB will be accelerated.

## Supporting information

S1 File Supplementary Tables S1 and S2, and Figure S1. (DOCX)
