# Supplementary material for: Evaluation of health effect on workers exposed to methyl bromide with prefrontal event-related potential
Source: PLoS One. 2025 Jul 30;20(7):e0328580. doi: 10.1371/journal.pone.0328580 (PMC12310017; doi:10.1371/journal.pone.0328580)

Supplementary Material

Evaluation of health effect on workers exposed to methyl bromide with prefrontal event-related potential

**Jungmi Choi^1^, Min-Goo Park^2*^**

^1^Human Anti-Aging Standards Research Institute, Uiryeong-gun, Gyeongsangnam-do, Republic of Korea

^2^Department of Bioenvironmental Chemistry, Jeonbuk National University, Jeonju, Republic of Korea

*** Correspondence:** Min-Goo Park: [pmg@korea.kr](mailto:pmg@korea.kr)

**Table S1. General work and ERP measurement processes of the subjects**

| Time | Fumigator | Inspector |
| --- | --- | --- |
| 08:00 | **Measure ERP indices**  **after stabilization** | - |
| 08:30 | - | **Measure ERP indices**  **after stabilization** |
| 09:00 | Move to working area | Prepare documents and travel to work area |
| 09:30 | Prepare MB injections  - Calculate MB dose  - Connect hose  - Seal container | Inspect plants  - Check documents and plants  - Open packing materials  - Visually inspect plants by cutting or sifting |
| 11:00 | Inject MB ^1^ | Oversee MB injections |
| 11:30 | Completion of MB injections | Complete overseeing MB injections |
| 12:00 | Lunch | Lunch |
| 13:30 | Degas MB  - Measure MB concentration  - Remove tape on containers  - Open containers  - Wait for MB concentration reduction (approximately 2hrs) | Confirm MB concentrations(~13:40)  Inspect plants (13:40~)  - Check document and plants  - Open packing materials  - Visually inspect plants by cutting or sifting |
| 15:30 | Travel back to office | Travel back to office |
| 16:00 | **Measure ERP indices**  **after stabilization** | Document findings |
| 16:30 | - | **Measure ERP indices**  **after stabilization** |

Subjects’ urine was collected just before the measurement of ERP indices. SDPTG indices and urinary Br- concentrations were measured in one or two subjects per group on the day of MB fumigation work.

^1^ MB fumigation was carried out at quarantine area in Busan port, MB was injected with 33-73 g/m3 on wood depending on temperature, and 64 g/m3 on oranges at 1℃.

**Table S2. The data on fumigators’ Sensory ERP indices and urinary bromide ion**

| **Subject-ID** | **Age** | **Bromide ion(μg/mg CRE)** | | **Latency (ms)** | | **Amplitude (㎶)** | |
| --- | --- | --- | --- | --- | --- | --- | --- |
|  |  | **Before** | **After** | **Before** | **After** | **Before** | **After** |
| Fumi-01 | 37 | 14.40 | 63.17 | 252 | 258 | 3.276 | 3.198 |
| Fumi-02 | 31 | 10.12 | 15.41 | 256 | 254 | 1.637 | 1.7415 |
| Fumi-03 | 47 | 9.26 | 19.18 | 264 | 268 | 2.656 | 2.54 |
| Fumi-04 | 51 | 4.21 | 6.54 | 264 | 270 | 3.583 | 4.26 |
| Fumi-05 | 39 | 2.07 | 8.41 | 250 | 256 | 1.1115 | 2.3905 |
| Fumi-06 | 42 | 2.20 | 2.71 | 270 | 270 | 3.1665 | 4.085 |
| Fumi-07 | 35 | 0.53 | 14.40 | 264 | 260 | 3.814 | 3.003 |
| Fumi-08 | 32 | 2.63 | 18.56 | 270 | 266 | 1.911 | 1.249 |
| Fumi-09 | 46 | 4.39 | 7.73 | 272 | 274 | 2.081 | 1.905 |
| Fumi-10 | 37 | 5.33 | 17.59 | 248 | 232 | 2.186 | 1.9325 |
| Fumi-11 | 39 | 2.37 | 18.47 | 264 | 258 | 3.5615 | 3.667 |
| Fumi-12 | 44 | 3.48 | 10.76 | 262 | 254 | 2.67 | 3.5255 |
| Fumi-13 | 59 | 2.54 | 19.08 | 268 | 268 | 2.2625 | 1.6135 |
| Fumi-14 | 44 | 6.34 | 1.68 | 260 | 262 | 0.9505 | 0.9535 |
| Fumi-15 | 41 | 4.02 | 13.99 | 272 | 264 | 3.246 | 3.5355 |
| Fumi-16 | 34 | 10.55 | 31.86 | 232 | 232 | 4.384 | 3.39 |
| Fumi-17 | 30 | 4.17 | 10.80 | 272 | 264 | 2.2835 | 3.2385 |
| Fumi-18 | 39 | 7.65 | 4.95 | 252 | 254 | 2.6315 | 2.581 |
| Fumi-19 | 32 | 5.18 | 4.91 | 302 | 248 | 2.1725 | 2.15 |
| Fumi-20 | 40 | 6.14 | 8.01 | 308 | 252 | 1.5875 | 2.0725 |
| Fumi-21 | 50 | 10.33 | 14.03 | 280 | 280 | 2.4435 | 2.065 |
| Fumi-22 | 28 | 4.23 | 11.24 | 264 | 256 | 2.7855 | 3.026 |
| Fumi-23 | 45 | 13.83 | 16.00 | 264 | 260 | 2.7045 | 2.2035 |
| Fumi-24 | 66 | 1.24 | 5.06 | 260 | 256 | 3.437 | 3.677 |
| Fumi-25 | 59 | 5.33 | 9.55 | 272 | 276 | 2.4915 | 2.736 |
| Fumi-26 | 56 | 7.87 | 35.85 | 284 | 284 | 2.522 | 2.067 |
| Fumi-27 | 50 | 1.63 | 1.33 | 258 | 264 | 2.3785 | 2.8625 |
| Fumi-28 | 43 | 6.58 | 7.90 | 258 | 308 | 2.407 | 1.8515 |
| Fumi-29 | 39 | 1.67 | 55.83 | 268 | 266 | 2.335 | 2.5565 |
| Fumi-30 | 29 | 2.89 | 11.82 | 252 | 254 | 2.399 | 2.916 |
| Fumi-31 | 61 | 11.58 | 46.01 | 276 | 274 | 0.558 | 1.134 |
| Fumi-32 | 50 | 7.47 | 34.57 | 274 | 278 | 1.847 | 1.4665 |

**Table S3. The data on inspectors’ Sensory ERP indices and urinary bromide ion**

| **Subject-ID** | **Age** | **Bromide ion(μg/kg)** | | **Latency (ms)** | | **Amplitude (㎶)** | |
| --- | --- | --- | --- | --- | --- | --- | --- |
|  |  | **Before** | **After** | **Before** | **After** | **Before** | **After** |
| Insp-01 | 58 | 0.51 | 2.56 | 256 | 236 | 0.5395 | 1.6415 |
| Insp-02 | 46 | 5.26 | 4.70 | 284 | 266 | 1.7625 | 1.8405 |
| Insp-03 | 29 | 2.21 | 2.93 | 258 | 246 | 3.484 | 3.6385 |
| Insp-04 | 49 | 5.22 | 2.25 | 252 | 244 | 2.664 | 4.05 |
| Insp-05 | 47 | 3.16 | 4.20 | 270 | 250 | 2.2735 | 2.805 |
| Insp-06 | 29 | 5.25 | 8.82 | 270 | 264 | 1.277 | 2.218 |
| Insp-07 | 53 | 2.60 | 2.47 | 276 | 270 | 2.011 | 1.9435 |
| Insp-08 | 21 | 4.34 | 14.22 | 266 | 262 | 2.029 | 2.7465 |
| Insp-09 | 35 | 4.52 | 6.35 | 274 | 266 | 1.1365 | 1.861 |
| Insp-10 | 47 | 11.25 | 9.23 | 272 | 264 | 3.1475 | 4.247 |
| Insp-11 | 33 | 0.21 | 0.41 | 264 | 258 | 1.778 | 2.816 |
| Insp-12 | 30 | 4.64 | 0.78 | 242 | 242 | 2.4265 | 2.4935 |
| Insp-13 | 28 | 0.59 | 0.66 | 254 | 250 | 3.702 | 3.7585 |
| Insp-14 | 29 | 3.80 | 0.58 | 246 | 238 | 2.1265 | 2.576 |
| Insp-15 | 36 | 7.48 | 4.42 | 248 | 242 | 2.722 | 2.891 |
| Insp-16 | 28 | 8.03 | 4.21 | 254 | 252 | 1.8805 | 3.129 |
| Insp-17 | 35 | 1.93 | 0.38 | 270 | 276 | 1.392 | 1.071 |
| Insp-18 | 20 | 5.92 | 6.29 | 268 | 268 | 2.0595 | 2.0755 |

**Figure S1. HPLC chromatogram**


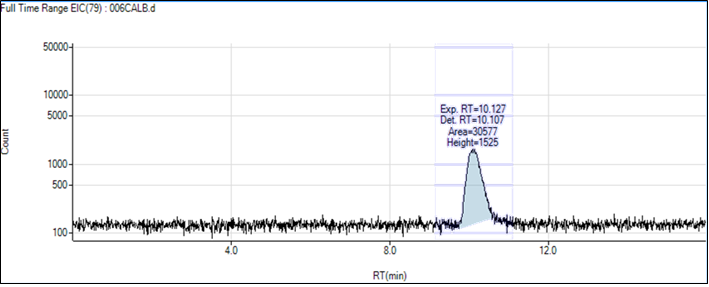

Supplement: S1 File — (DOCX) [file pone.0328580.s001.docx]
